# Higher Frequency of SARS-CoV-2 RNA Shedding by Cats than Dogs in Households with Owners Recently Diagnosed with COVID-19

**DOI:** 10.3390/v16101599

**Published:** 2024-10-11

**Authors:** Michele Lunardi, Felippe Danyel Cardoso Martins, Emanuele Gustani-Buss, Roberta Torres Chideroli, Isabela Medeiros de Oliveira, Kamila Chagas Peronni, David Livingstone Alves Figueiredo, Alice Fernandes Alfieri, Amauri Alcindo Alfieri

**Affiliations:** 1 Laboratory of Animal Virology, Department of Veterinary Preventive Medicine, Universidade Estadual de Londrina, Londrina 86057-970, Brazil; michelelunardi@gmail.com (M.L.); aalfieri@uel.br (A.F.A.); 2 Multi-User Animal Health Laboratory, Molecular Biology Unit, Department of Veterinary Preventive Medicine, Universidade Estadual de Londrina, Londrina 86057-970, Brazil; felippew@gmail.com; 3 Post Graduate Program in Animal Health and Production, Department of Agrarian Sciences, University Pitagoras Unopar, Arapongas 86702-670, Brazil; 4 Post Graduate Program in Animal Science, Department of Veterinary Preventive Medicine, Universidade Estadual de Londrina, Londrina 86057-970, Brazil; roberta.tchideroli@gmail.com; 5 Department of Microbiology, Immunology and Transplantation, Rega Institute, KU Leuven—University of Leuven, Box 1030, 3000 Leuven, Belgium; emanuele.gustani-buss@kuleuven.be; 6 Institute for Cancer Research, IPEC, Guarapuava 85100-000, Brazil; isamoliveira20@gmail.com (I.M.d.O.); kamila@ipec.org.br (K.C.P.); davidlafigueiredo@gmail.com (D.L.A.F.); 7 Department of Medicine, Midwestern Parana State University—UNICENTRO, Guarapuava 85040-167, Brazil; 8 National Institute of Science and Technology for Dairy Production Chain (INCT–LEITE), Universidade Estadual de Londrina, Londrina 86057-970, Brazil

**Keywords:** one health, zoonoses, pets, coronavirus, severe acute respiratory syndrome coronavirus 2, RT-qPCR, next generation sequencing

## Abstract

Studies have demonstrated the susceptibility of companion animals to natural infection with SARS-CoV-2. Using quantitative reverse transcription polymerase chain reaction and sequencing analyses, this study investigated SARS-CoV-2 RNA excretion in pets in households with infected owners. Oropharyngeal and rectal swabs were collected from dogs and cats in Parana, Southern Brazil, between October 2020 and April 2021. Viral RNA was detected in 25% of cats and 0.98% of dog oropharyngeal swabs; however, systemic, respiratory, and gastrointestinal signs were absent. Complete viral genomes belonged to the Gamma lineage. Phylogenetic analyses indicated that pet samples were probably derived from human-positive cases in Parana. Viral excretion in the oropharynx was more frequent in cats than in dogs. Mutations in the S protein characteristic of Gamma strains were present in all sequenced SARS-CoV-2 strains. The receptor-binding domain of these Brazilian strains did not show any additional mutations not reported in the Gamma strains. Mutations in NSP6, NSP12, and N proteins previously mapped to strains that infect deer or minks were detected. This study highlights the importance of actively monitoring the SARS-CoV-2 strains that infect pets with continued viral exposure. Monitoring genetic changes is crucial because new variants adapted to animals may pose human health risks.

## 1. Introduction

Coronaviruses are a large group of viruses that belong to the family *Coronaviridae*. Coronaviruses in the subfamily *Orthocoronavirinae* are classified into four genera: *Alphacoronavirus*, *Betacoronavirus*, *Deltacoronavirus*, and *Gammacoronavirus*. In late 2019, a new coronavirus, severe acute respiratory syndrome coronavirus 2 (SARS-CoV-2), was identified in Wuhan, China, as the causative agent of the novel coronavirus disease 2019 (COVID-19), which affected public health systems worldwide, causing over 775 million cases and approximately 7 million deaths worldwide [1]. SARS-CoV-2 is collectively classified under the *Betacoronavirus* genus, along with SARS-CoV, which emerged in 2002 [2].

As of June 2024, data obtained by the World Health Organization (WHO) indicated that 37,511,921 reported cases and 702,116 deaths have been confirmed in Brazil [1]. In 2020, the COVID-19 epidemic in Brazil was initially characterized by the circulation of lineages B.1.1.28 and B.1.1.33, which were first replaced by the emergence of lineage P.2 in the Rio de Janeiro state, probably in late 2020, becoming the dominant lineage in several Brazilian states from November 2020 to January 2021 [3,4]. In November 2020, a second major lineage replacement occurred in the Amazonas state, with the emergence and dissemination of lineage P1, currently known as variant of concern (VOC) gamma, which became the most prevalent lineage in all Brazilian regions by early 2021 [5].

Currently, the most probable hypothesis for the origin of SARS-CoV-2 is supported by a growing body of scientific evidence that points to natural zoonotic emergence, which is likely associated with the wildlife trade and/or fur farming [6]. Since coronaviruses of bat origin have been identified and shown to be highly similar to SARS-CoV-2, bats have been suggested to be its natural host. Among the bat coronaviruses already characterized, the virus designated Banal-20-52, originally detected in a *Rhinolophus* bat in Laos, shares the highest identity with SARS-CoV-2 across the entire genome, exhibiting 96.8% nucleotide sequence similarity. A comparison of the amino acid sequence comprising the receptor-binding domain (RBD) of the spike glycoprotein showed that Banal-20-52 was almost identical to the corresponding region of SARS-CoV-2 [7]. However, as direct transmission of the virus from bats to humans is less likely, it has been suggested that the pathogen was introduced through an intermediate terrestrial mammal host, such as the pangolin (*Manis javanica*) [8,9].

Since the emergence of SARS-CoV-2, natural infections have been described in several animal hosts, including those classified in the orders Primate, Pilosa, Rodentia, Carnivora, Sirenia, Artiodactyla, and Perissodactyla, in 44 countries [10]. SARS-CoV-2 infection has been reported in both wild and domestic members of the Felidae family and in domestic dogs of the Canidae family [11]. Since the beginning of the COVID-19 pandemic, several studies have demonstrated the susceptibility of domestic cats and dogs to natural infection with SARS-CoV-2. This infection is mainly associated with contact with infected owners [12,13,14,15]. Clinical manifestations of varying severity have been observed in both hosts, ranging from asymptomatic infections to respiratory diseases and severe outcomes in the presence of comorbidities [14,16,17,18,19,20]. Moreover, cats have been reported to be more susceptible than dogs to infection, and the airborne transmission of the viral agent from experimentally infected cats to susceptible cats has been documented [21].

An important aspect of the SARS-CoV-2 infection is the capacity of the virus to be transmitted from humans to other animal species. Thus, cases wherein infected zookeepers pass the virus to captive tigers and lions at the New York Zoo have been classified as zooanthroponose or reverse zoonosis. However, cases wherein strains with animal sequence signatures from naturally infected minks are transmitted to farm workers have been classified as an anthropozoonose [22,23]. Remarkably, a high rate of evolutionary change has been observed in specific animal species following SARS-CoV-2 infection, indicating a certain degree of host adaptation [24].

Using quantitative reverse transcription polymerase chain reaction (RT-qPCR) and sequencing, the present study aimed to investigate SARS-CoV-2 RNA excretion in the biological fluids of companion animals sharing households with owners who were recently diagnosed with COVID-19 through active surveillance during the second COVID-19 wave in Brazil between October 2020 and April 2021.

## 2. Materials and Methods

### 2.1. Animal Enrollment

The study inclusion criterion was domestic dogs or cats in a household with at least one human diagnosed with COVID-19 for up to 10 d prior to pet enrollment. Other factors, such as age, sex, breed, vaccination, and clinical status, were not considered. Humans who recently tested positive for SARS-CoV-2 via RT-qPCR were contacted during quarantine and asked if they wanted to enroll their pets in the study. Interested pet owners provided an informed consent form for their animals to participate in the study. This study was approved by the Ethics Committee for Animal Use of Universidade Estadual de Londrina (Protocol No. 043.2020).

### 2.2. Sampling

Clinical samples, particularly oropharyngeal and rectal swabs, were collected from dogs and cats living with owners recently diagnosed with COVID-19 in Londrina, Parana state, southern Brazil, between October 2020 and April 2021. The swabs used were rayon-tipped applicators and were immediately placed in 3 mL of phosphate-buffered saline. All samples were kept at 0 °C during collection and stored at −80 °C until processing. During sample collection, the owners of the sampled animals completed an epidemiological questionnaire that covered questions regarding signalment (age, sex, and breed), presence of clinical signs (systemic, respiratory, or gastrointestinal alterations), vaccination status, the interval between positive human test results and pet sampling, and level of contact between the owner and companion animal.

### 2.3. Nucleic Acid Extraction

Nucleic acid was purified from the oropharyngeal and rectal swab samples using an EXTRACTA 32 kit (MVXA-P016 FAST) (Loccus, Cotia, Brazil) in an automated extractor (Loccus), following the manufacturer’s instructions. To monitor RT-qPCR amplification and inhibition, 2 μL (20,000 copies per sample) of VetMAX Xeno Internal Positive Control RNA (Applied Biosystems, Waltham, MA, USA) was used.

### 2.4. RT-qPCR

The RT-qPCR assay targeted a partial fragment of the SARS-CoV-2 nucleocapsid gene (region N1) in nucleic acids extracted from oropharyngeal and rectal swab samples. The assay was performed on an ABI 7500 Fast Real-Time PCR System (Applied Biosystems) using the “auto baseline” setting to determine fluorescence baselines. The AgPath-ID One-Step RT-PCR and VetMAX Xeno Internal Positive Control—VIC Assay kits (Applied Biosystems) and primers and probes following the CDC SARS-CoV-2 protocol were used [25]. Each reaction had a total volume of 15 μL, containing 5 μL of purified nucleic acid, 7.5 μL of 2× RT-PCR buffer, 500 nM of each primer, 125 nM probe, 0.6 μL of 25× RT-PCR enzyme mix, and 0.6 μL of the Xeno VIC Primer Probe Mix (Applied Biosystems). The thermocycling conditions of the reaction were as follows: 50 °C for 10 min for reverse transcription, 95 °C for 10 min for transcriptase inactivation, and 40 cycles of 95 °C for 15 s and 60 °C for 1 min.

RNA extracted from a human biological sample previously confirmed to contain SARS-CoV-2 RNA was used as a positive control, and ultrapure water was used as a negative control for RT-qPCR.

### 2.5. Whole-Genome Sequencing and Clade Assignment

For the whole-genome sequencing of the SARS-CoV-2 strains present in domestic cats and dogs, viral RNA was extracted from respiratory fluids using a QIAamp Viral RNA Mini Kit (Qiagen, Hilden, Germany), following the manufacturer’s instructions. A library was prepared following the Illumina COVIDSeq kit protocol (Illumina, San Diego, CA, USA), and sequencing was performed on a NovaSeq 600 platform (Illumina) using the NovaSeq 6000 SP Reagent kit v1.5 (200 cycles) (Illumina).

Genome assembly was performed using the Genome Detective application [26] to obtain consensus sequences from Illumina reads. The pipeline included trimming and filtering using Trimmomatic [27]; assessment of the quality of reads before and after filtering using FASTQ [28]; read classifications using DIAMOND to select the viral sets containing the protein cluster to be annotated on the Swissprot database; *de novo* assembly using metaSPAdes; and searching for a candidate reference genome (NC_045512.2) against the NCBI RefSeq virus database using Blastx and Blastn. To optimize and define the best global alignment, Advanced Genome Aligner [29] was implemented to generate the contigs, which were saved in the FASTA file format.

The pangolin web application (https://cov-lineages.org (accessed on 22 July 2024)) [30], developed for the phylogenetic classification of global outbreak lineages, was used to assign the lineages. Clade assignment and assessment of the quality of complete sequences were performed using the Nextstrain platform (https://clades.nextstrain.org (accessed on 22 July 2024)) [31]. The Ultrafast Sample Placement on Existing Trees (UShER) web application, designed by the University of California, Santa Cruz (https://genome.ucsc.edu/cgi-bin/hgPhyloPlace (accessed on 22 July 2024)), was used to assess evolutionary relationships using a phylogenetic tree with over 16 million available genomes [32,33]. The sequence data were registered with the Global Initiative on Sharing All Influenza Data (GISAID) using the following numbers: EPI_ISL_19344592, EPI_ISL_19344593, EPI_ISL_19344594, and EPI_ISL_19344595.

Maximum-likelihood phylogenetic analysis was performed using IQ-TREE2, specifying the model finder function, which selected the General Time Reversible (GTR) substitution model with empirical base frequencies (+F), invariant sites (+I), and three categories of rate variation (+R3), with a phylogeny test of 1000 replicates for ultrafast bootstrap and SH-aLRT support procedure analysis. The database was constructed based on the sequences available in GISAID, covering the period of the circulation of samples comprising 2010 sequences from five Brazilian regions, including the Parana state.

## 3. Results

Between October 2020 and April 2021, during the second COVID-19 wave in Brazil, when over 400,000 human cases were registered in Brazil, 122 pets from 80 households participated in the study. Among them, 102 dogs of both sexes and 20 different breeds, ranging in age from 2 months to 15 years, were included. Additionally, 20 cats of both sexes and mostly shorthaired mixed breeds, ranging in age from 2 months to 4 years, were sampled for a molecular investigation of SARS-CoV-2 infection.

In this study, RT-qPCR was used to analyze oropharyngeal swab samples collected from cats and dogs living with owners who were recently diagnosed with COVID-19. Viral RNA was detected in 25% (5/20) and 0.98% (1/102) of the swabs from cats and dogs, respectively. None of the rectal swabs from cats or dogs tested positive following RT-qPCR. The epidemiological data of animals that shed SARS-CoV-2 RNA via oral secretion are presented in Table 1.

During sample collection, clinical examinations were conducted to record any noticeable signs that were previously observed in cats and dogs infected with SARS-CoV-2. Systemic, respiratory, or gastrointestinal signs were not present in the cats shedding viral RNA through the upper respiratory mucosa, while one dog had ocular secretions and an enlarged popliteal lymph node (Table 1).

All SARS-CoV-2 RNA-positive companion animals identified in this investigation lived in a household with owners with a recent history of COVID-19, confirmed molecularly via RT-qPCR. The period between the laboratory confirmation of infection with SARS-CoV-2 in the human household member and the time of specimen collection from their respective pets was less than 10 d.

Of the 80 pet households evaluated, 5 (6.25%) had at least one dog or cat that shed viral RNA through their upper respiratory mucosa. Interestingly, in one of the multipet households, a dog and cat tested positive for viral RNA in RT-qPCR, confirming recent exposure (Table 1).

**Figure 1:**
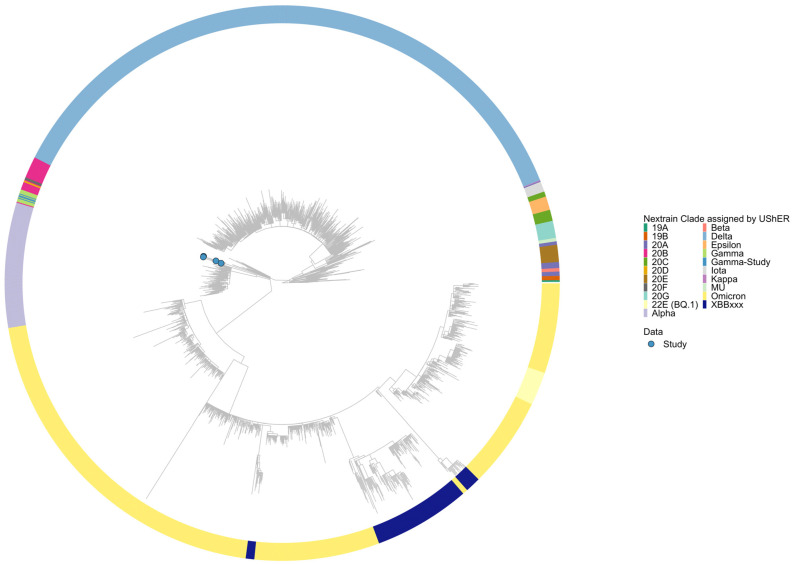
Phylogenetic reconstruction of the four Brazilian SARS-CoV-2 strains sequenced from infected companion animals based on complete genome sequences. The evolutionary history was inferred using UShER, in which over 16,000,000 high-coverage whole-genome SARS-CoV-2 sequences from the public databases GISAID, GenBank, COG-UK, and CNCB were included in the analyses (updated 31 May 2024). The phylogenetic placement of the 077_cat, 103_cat, 109_cat, and 110_dog Brazilian strains (highlighted in light blue) in the subtree containing 2004 random sequences from the global maximum parsimony based-phylogenetic tree is shown in the radial layout.

Complete viral genomes from the respiratory tract samples of four of the six RT-qPCR-positive animals were sequenced. Four complete genomes were found to belong to the Gamma lineage (P.1). The lineage and clade classifications of all strains were confirmed. A phylogenetic tree containing over 16,000,000 complete high-coverage SARS-CoV-2 sequences from public sequence databases including GISAID, GenBank, COG-UK, and CNCB (Figure 1) showed that all the strains were grouped in the same cluster.

The nearest neighboring public sequence for each genome was assessed through UShER. The Brazilian strain 077_cat is most closely related to isolate SARS-CoV-2/human/BRA/SC-NXBS1548GENOV45615126547/2021, which was obtained from an infected human in the Santa Catarina state in June 2021 (GenBank Accession No.: ON576304). For strains 103_cat and 109_cat, the most similar complete sequences are isolates SARS-CoV-2/human/BRA/PR-NVBS995GENOV44905645799/2021 (GenBank Accession No.: ON577406) and SARS-CoV-2/human/BRA/PR-NVBS3858GENOV827809279051/2021 (GenBank Accession No.: ON576613), respectively, which were sequenced in June and July 2021 from infected humans in the state of Parana. For the Brazilian strain 110_dog, SARS-CoV-2/human/BRA/RJ-NXBS1856GENOV827659789175/2021 (GenBank Accession No.: ON576796), sequenced in June 2021 from an infected human in Rio de Janeiro, was the closest neighboring complete sequence.

**Figure 2:**
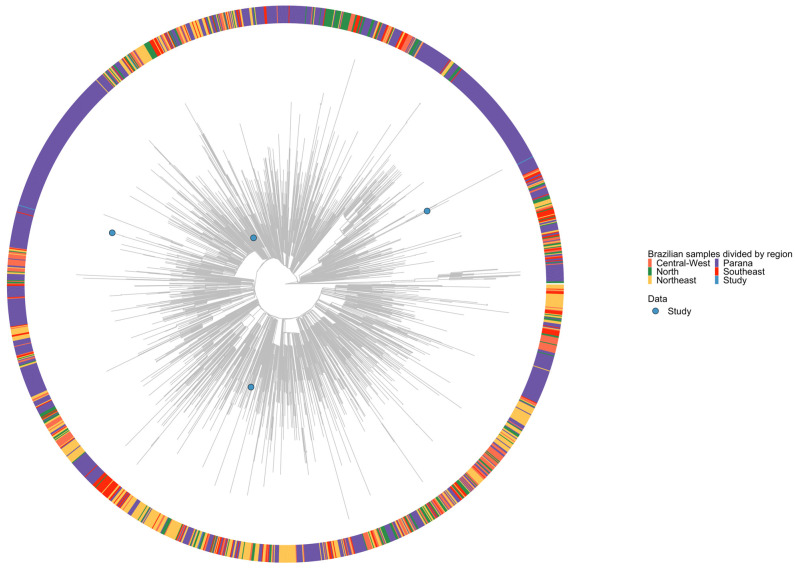
Maximum-likelihood phylogenetic analysis of SARS-CoV-2 genome sequences from 2014 genomes representative of all Brazilian regions obtained from GISAID from December 2020 to September 2021. The phylogenetic placement of study samples is highlighted in light blue in both categories.

In addition, a maximum-likelihood phylogenetic tree of the Gamma variant of SARS-CoV-2, which included 2014 sequences from the Parana state (900 genomes) and all other Brazilian regions (1114 genomes), indicated that the pet samples were probably derived from human-positive cases in the majority of Parana, with strong support (80% of UF-bootstrapping replicates). Phylogenetic analysis revealed that the genome most closely related to 077_cat belonged to a case confirmed in Parana in July 2021 (hCoV-19/Brazil/PR-NVBS3844GENOV827795420208/2021|EPI_ISL_4414392|2021-07-03). Similarly, 103_cat clustered with a genome isolated from Parana in June 2021 (hCoV-19/Brazil/PR-FIOCRUZ-59460/2021|EPI_ISL_8005416|2021-06-26). For 109_cat, the most similar complete genome was that of an isolate from the same city of origin (Londrina, Parana, Brazil) obtained in April 2021 (hCoV-19/Brazil/PR-IPEC_VIGCV19_LON_0599/2021|EPI_ISL_12425597|2021-04-04). Additionally, 110_dog clustered with a genome collected from isolates in the southern region of Rondonia state, northern region, in July 2021 (hCoV-19/Brazil/RO-IAL 6650/2021|EPI_ISL_7982772|2021-07-05) (Figure 2).

**Figure 3:**
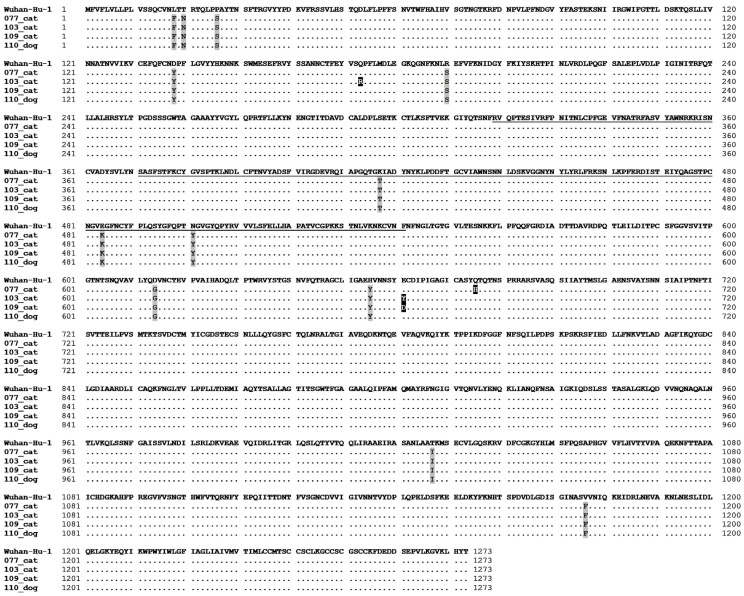
Multiple sequence alignment of amino acid sequences of the SARS-CoV-2 spike protein of the reference strain Wuhan-Hu-1 (GenBank Accession No.: NC_045512) and the four Brazilian strains sequenced from companion animals in this study. Amino acid residues different from the corresponding ones in the Wuhan-Hu-1 strain and characteristic of the Gamma lineage (P1) are highlighted in gray. Mutational changes observed in the Brazilian strains are shown in a white font and highlighted in black. The region marked with black lines indicates the RBD.

The Spike (S) gene sequences from the four SARS-CoV-2 strains sequenced in this study shared high identities among themselves, ranging from 99.8% to 99.9% at the nucleotide level and 99.7% to 99.9% at the amino acid level. Compared with the reference strain Wuhan-Hu-1 from lineage B, the identities ranged from 99.5 to 99.6% at the nucleotide level and 98.9 to 99% at the amino acid level. Mutations in the amino acid sequence of the S protein of the Gamma strains of SARS-CoV-2 characteristic of this particular VOC were detected in all four strains analyzed in this study. The specific locations of these mutations within the S protein are shown in Figure 3.

## 4. Discussion

This investigation showed that a proportion of domestic cats (25%) and dogs (0.98%) living in a household with humans who were recently diagnosed with COVID-19 were infected and shed SARS-CoV-2 RNA through oral fluids. Similar findings were also reported by several other previous studies conducted worldwide [34,35,36,37].

The findings of a previous study [13] conducted in the United States showed that domestic cats in households with confirmed human infections had a higher rate of SARS-CoV-2 infection than domestic dogs in similar households. The study revealed PCR positivity rates of 17.6% (3/17) for domestic cats and 1.7% (1/59) for domestic dogs. These numbers were observed in 10.3% (4/39) of the households evaluated [13]. Similarly, a longitudinal prospective study conducted in Northeastern Brazil found that 15% (2/15) of cats and none of the dogs exposed to infected tutors tested positive for SARS-CoV-2 using RT-qPCR [38]. A multicenter study conducted in four geographical regions of Brazil, which included pets in households with infected humans, reported that 11% of dogs shed SARS-CoV-2 RNA, whereas 20.83% of cats were infected [39]. In 2020, researchers investigated companion animals owned by individuals who tested positive for SARS-CoV-2 in Rio de Janeiro, Brazil [12]. In contrast to our findings, the study revealed that among the animals evaluated, 28% of dogs and 40% of cats from 47.6% of households with at least one human recently diagnosed with COVID-19 tested positive for SARS-CoV-2 infection [12].

In the present study, we found that the detection frequency of SARS-CoV-2 RNA in the respiratory tract was higher in cats than in dogs. This finding is in agreement with other studies and may suggest that domestic cats are more susceptible to SARS-CoV-2 infection than domestic dogs, especially in households with confirmed human cases of COVID-19. Similar findings were reported in past epidemiological surveys [12,13,35,36,38,40]; however, additional investigations encompassing a large number of companion animals with continued exposure to the virus are necessary to confirm this observation. In addition, previous studies have shown that domestic cats are highly susceptible to SARS-CoV-2 infection and can horizontally transmit the virus to other cats through direct or indirect contact [21,41,42,43]. In contrast to what has been observed in domestic cats, laboratory challenge studies have shown evidence of a lower susceptibility of domestic dogs to viral infection, accompanied by limited replication [21,41].

A noteworthy finding in the current study was the identification of SARS-CoV-2 excretion using RT-qPCR in the respiratory mucosal fluid of a dog and cat that cohabited a household with a confirmed human infection, confirming a recent exposure (Table 1). In a study conducted in the USA, in two multipet houses where both cats and dogs lived with humans with COVID-19, the cats tested positive, whereas the dogs tested negative for SARS-CoV-2 RNA via RT-qPCR [13]. In addition, in Rio de Janeiro, Brazil, a cat and dog living with an infected owner tested positive for the virus via RT-qPCR and sequencing [12]. This was also observed in Arizona, USA, where a cat and dog living with a symptomatic owner who tested positive for SARS-CoV-2 tested positive for the virus. Both pets remained asymptomatic despite close contact with their owner [15].

In the present investigation, using RT-qPCR, SARS-CoV-2 RNA was absent in rectal swabs, even in dogs and cats that tested positive for the virus in respiratory samples. In contrast, in previous surveys, SARS-CoV-2 was detected in fecal samples but only in a small proportion of infected dogs and cats [12,13,38,39].

Clinical examinations did not reveal the occurrence of overt signs previously associated with viral infection in companion animals that shed SARS-CoV-2 RNA through the respiratory mucosa. Several studies conducted worldwide have investigated the occurrence of clinical gastrointestinal or respiratory signs in pets living with humans with COVID-19. The results are diverse, with some reporting no clinical signs in pets and others showing signs that vary in frequency and intensity [12,13,40,44,45]. A multicenter study conducted in Brazil involving pets with owners who were recently infected with SARS-CoV-2 revealed that 40% of infected cats and 11.11% of infected dogs were symptomatic for respiratory illness [39]. However, a recent study employing genomic sequencing showed that an 8-year-old male cat co-infected with feline leukemia virus suffered a multisystemic and lethal SARS-CoV-2 infection caused by a viral strain classified as variant P.1 (Gamma) [19].

SARS-CoV-2 infection in domestic cats and dogs was diagnosed in the present study using RT-qPCR, targeting a partial fragment of the gene that encodes the nucleocapsid protein. This molecular technique has been routinely employed for the laboratory diagnosis of COVID-19 in humans and has shown success in diagnosing this viral infection in the companion animals evaluated herein and in previous investigations [12]. Several studies using serological tests based on the conserved viral protein N to diagnose SARS-CoV-2 infection in dogs and cats have shown inconsistent results compared to those using different serological assays. This could be due to cross-reactivity with other endemic coronaviruses present in these animal species, such as canine and feline coronaviruses [46,47]. Also noteworthy is the higher frequency of viral shedding detected by RT-PCR compared with that of seropositivity for neutralizing antibodies against SARS-CoV-2 in cats and dogs [13].

In this study, genetic analyses showed that the characteristic mutations of Gamma strains within the N-terminal domain (NTD), namely L18F, T20N, P26S, D138Y, and R190S, and those located in the RBD of SARS-CoV-2, namely K417T, E484K, and N501Y, were present in the four sequenced strains (Figure 3). The RBD of the S1 subunit of SARS-CoV-2 is responsible for angiotensin-converting enzyme 2 recognition and binding [48]. The RBD of the Brazilian strains did not show any additional mutations that have not been previously reported in Gamma strains. However, their genomes exhibited the following mutational changes in the S protein: Q173R within the NTD of the SARS-CoV-2 103_cat strain; E661Y and E661D in the S1 subunit of the 103_cat and 109_cat strains, respectively; and Q675H in the S1 subunit of the 077_cat strain (Figure 3).

Additionally, a comparison of the genomes of the Brazilian strains with that of the reference strain Wuhan-Hu-1 revealed amino acid changes along different open reading frames: S408F (103_cat strain), A31V, and D449N (077_cat strain) in NSP2; S370L and K997Q (all four strains), V207L, P968S, T1002I, and T1677I (077_cat strain) in NSP3; L260F (077_cat strain) in NSP6; P323L (all four strains) in NSP12; E341D (all four strains) and T351A (077_cat strain) in NSP13; S253P (all four strains) in NS3; P71S (077_cat strain) in E; E92K (all the strains), F120V and I121L (109_cat strain) in NS8; and A35T (103_cat strain), P80R, R203K, and G204R (all four strains) in N. In previous investigations, mutations L260F in NSP6, P323L in NSP12, and R203K and G204R in the N protein were also mapped to SARS-CoV-2 strains that infect deer or minks, demonstrating that these amino acid variants may represent novel nonhuman, animal-associated SARS-CoV-2 mutations [49]. However, the sequencing of additional strains infecting animal species, including domestic cats and dogs from diverse geographic areas, is recommended to confirm these findings.

The structural protein S of SARS-CoV-2 is crucial for viral infection because it is necessary for target recognition, cellular entry, and endosomal escape [50]. The positive selection of mutational changes in the spike protein of SARS-CoV-2 has led to the emergence of novel variants with increased infectivity. Thus, monitoring spike protein alterations, particularly in SARS-CoV-2 strains that infect animal species, is important to assess their evolution. Viral evolution in nonhuman species may increase evolutionary rates and allow for the selection of new mutations with increased transmission and virulence in humans [51,52,53].

Currently, the transmission of SARS-CoV-2 from infected individuals to pets is supported by research data [12,34,36,45,54]. Previous research has shown that dogs and cats sharing a household with infected owners have a higher risk of being infected with the virus, indicating that this viral agent may be transmitted from humans to pets more frequently than previously thought [55]. However, the current scientific evidence does not support the epidemiological role of domestic cats and dogs in transmitting SARS-CoV-2 to humans, as these animals appear to be dead-end hosts of the virus [42,56,57,58].

A limitation of our investigation is that clinical samples from owners recently diagnosed with COVID-19 by RT-qPCR, and sharing households with the infected pets, were not available for sequencing, which prevented the comparison between SARS-CoV-2 strains shed by pets and those excreted by their respective owners. Similarly, it was not possible to define the number of SARS-CoV-2-positive humans living in each household as well as the period of their viral shedding while interacting with the companion animals. Nonetheless, phylogenetic analyses employing complete genome sequences classified as part of the Gamma lineage and derived from human patients living in Brazil revealed that most pet strains were highly similar to human-positive cases that occurred in the Parana state between April and July 2021.

The information presented in this study and similar surveys highlights the importance of actively monitoring SARS-CoV-2 strains that infect pets with continued exposure to the virus. Monitoring genetic changes is crucial as new variants adapted to animals could potentially pose a risk to human health. Therefore, additional studies should be conducted in animals to monitor the mutations that may arise during SARS-CoV-2 infection. Moreover, individuals suspected of or confirmed to have COVID-19 should avoid close contact with their pets, including sharing a bed and/or food and kissing, during the recommended quarantine period [37,38]. Therefore, wearing masks when handling these animals has been suggested [12]. The implications of SARS-CoV-2 infection in companion animals for the public and animal health may be significant. However, data from epidemiological investigations of pets that were consistently exposed to people with COVID-19, regardless of their clinical status, are limited.

## Figures and Tables

**Table 1:** Epidemiological and clinical data of the six pets infected with SARS-CoV-2 between December 2020 and April 2021. The pets shared households with at least one confirmed case of a human recently diagnosed with COVID-19 in the Londrina municipality, Southern Brazil.

Animal ID	Household ID	Species	Breed	Sex ^a^/Age	Collection Date	Neighborhood Access	Close Contact with Tutor	Period between Sampling and Onset of Owner’s Symptoms	Clinical Signs at Visit	RT-qPCR Positive Sample	Ct Valeu N1	GISAID Accession Number
063	A	Cat	Mixed	F/3 y old	14 December 2020	Indoor	No	8 d	Asymptomatic	Oropharyngeal Swab	33	Not sequenced
077	B	Cat	Mixed	F/6 m old	13 January 2021	Indoor/Outdoor	Yes	12 d	Asymptomatic	Oropharyngeal Swab	35.6	EPI_ISL_19344592
103	C	Cat	Mixed	M/1 y old	9 April 2021	Indoor/Outdoor	Yes	9 d	Asymptomatic	Oropharyngeal Swab	31.4	EPI_ISL_19344593
109	D	Cat	Mixed	F/7 m old	15 April 2021	Indoor/Outdoor	Yes	9 d	Asymptomatic	Oropharyngeal Swab	29	EPI_ISL_19344594
110	D	Dog	Mixed	M/1 y old	15 April 2021	Indoor/Outdoor	Yes	9 d	Ocular secretion/Enlarged popliteal lymph node	Oropharyngeal Swab	35.2	EPI_ISL_19344595
115	E	Cat	Mixed	F/<1 y old	18 April 2021	Indoor/Outdoor	Yes	10 d	Asymptomatic	Oropharyngeal Swab	32.5	Not sequenced

^a^ F: female; M: male.

## Data Availability

The original data presented in the study are openly available in GISAID using the following numbers: EPI_ISL_19344592, EPI_ISL_19344593, EPI_ISL_19344594, and EPI_ISL_19344595.
